# Abundance across geographical species ranges and the rare edge hypothesis

**DOI:** 10.1098/rspb.2024.1874

**Published:** 2024-12-04

**Authors:** Jason P. Sexton

**Affiliations:** ^1^ Department of Life and Environmental Sciences, University of California, Merced, CA 95343, USA

**Keywords:** range limits, abundance, geographical range, community science, range dynamics

Species geographical ranges have long fascinated biogeographers. Do species ranges have universal properties that provide predictive relationships or is each species’ range idiosyncratic and unique in its essence? Perhaps something in between. One question that has been straightforward to ask, but complicated to answer, is whether species abundances (i.e., population occurences, sizes, density of individuals, etc.) peak in central areas of species ranges, also known as the abundant centre hypothesis (ACH) [1]. If true, the ACH would have myriad ramifications for ecological and evolutionary theory, phenomena and practice, including predicting regions of species ranges with the highest diversity, conservation value, evolutionary rates and dispersal patterns, etc. In this issue, Martin *et al*. [2] ask this question and frame a related but new question and hypothesis: are peripheral areas of species ranges rare in species abundances? Examining species range patterns of abundance using a highly robust dataset of North American birds and a powerful modelling approach, Martin *et al*. offer compelling evidence for this ‘rare edge hypothesis’ (REH). They found consistently lower bird relative abundance and occupancy near species range edges in nearly all cases examined. Additionally, abundance most often peaked not at the strict centres of species ranges, but somewhere in the interior or inner half of ranges.

Although it has been widely invoked, the ACH has also been questioned owing to doubts about its universality as a rule, as many studies have examined it and found inconsistent results over the past half century (e.g. [3–5]). It is challenging to evaluate hypotheses like the ACH and the REH. First, it is challenging to quantify abundance across a species’ entire geographical range, especially for wide-ranging species, which can cover a continent or more. Different abundance patterns can appear similar depending on sampling density and coverage (figure 1), and so robust and even sampling is necessary to verify different patterns. Second, there are many ways to quantify abundance, including the frequency of occurrence, the number of ‘populations,’ the density of individuals, etc. [6]. Which type of abundance, if not all, should be expected to conform to such patterns if they exist? Third, estimates of abundance are further complicated by the great diversity of species, with such varied life histories, modes and types of dispersal, mating systems, life spans, etc. Fourth, there are many methods to define, analyse and test for spatial patterns [5], including whether the ‘centre’ refers to the exact (strict) centre, or to interior, ‘core’ regions of species ranges [7]. Finally, defining central and non-central areas of species ranges and their edges can be challenging, and sampling between central and edge regions is rarely even [2].

**Figure 1.**
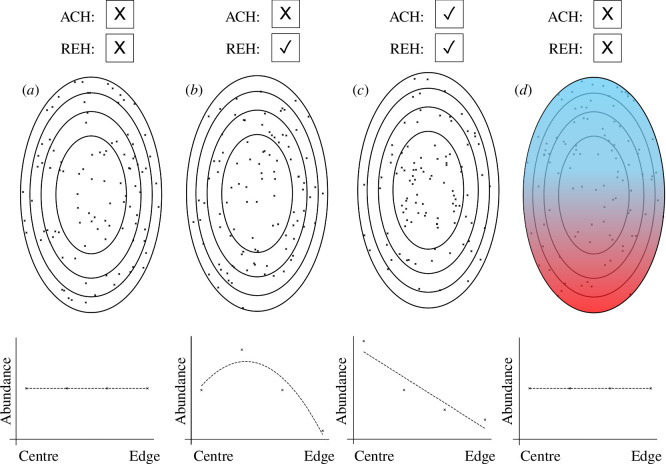
Hypothetical species ranges vary in abundance patterns. Each range has 100 populations distributed across four regions of equal area, from a central area to the outermost edge area. The graphs below each range show the corresponding population abundance patterns within each area, oriented from centre to edge. Check boxes above each range indicate whether each range conforms (✓) or does not conform (X) to the strict abundant centre hypothesis (ACH) and the rare edge hypothesis (REH). (*a*) A species range with equal abundance across all four regions. (*b*) A species range with lowest abundance at the edge and greatest abundance in an interior region but not in the strict centre. (*c*) A species range with greatest abundance in the centre and declining abundance towards the edge. (*d*) A species range with shifting abundance under climate warming, with warmer regions represented in red, and colder regions represented in blue. The colder (upper) half of the range has twice the abundance of the warmer half, and abundance declines further in the warmer half of the range from the centre to the rear edge. Nevertheless, each of the broader four areas of the range still have equal abundance in this scenario and so it is important to distinguish patterns between different climate ends of species ranges.

Martin *et al*. proposed the REH and provided multiple tests of it and the ACH using some of the best available data on bird species abundance patterns to date. The results were strongly in agreement with the REH, even when various measures and considerations were taken. Using the publicly available eBird dataset of breeding birds of North America—collected mainly through community scientists—gave a very large dataset of occurrences across many bird species ranges from which relative abundance could be modelled. Birders themselves seem to be abundant in North America and provide relatively reliable data, regardless of where in the given species range a report comes from. Birders often report on multiple species of the bird community at a given location, each occurrence in a different relative area of a given species’ respective range. This provides large occurrence datasets, and presumably with little bias for a species in question, although bird species do differ markedly in their difficulty of identification. Moreover, other potential issues exist, including the skill level of observers, motivations for a given report (e.g. providing a comprehensive survey of the avifauna, or focusing on a particular rarity/speciality in the area), time spent observing, time of day of observations, etc. Nevertheless, there have been notable advancements in the statistical models that allow the team at eBird to address errors in the raw observations and provide the geographically complete, modelled abundance maps that Martin *et al*. used for their study. Martin *et al*. examined eBird data for 109 bird species from one year (2018) in various ways, including analyses where the lack of occurrence (‘zeros’) was removed, analyses comparing oceanic versus terrestrial range edges and analyses using different criteria to define edge or central regions of species distributions (i.e. based on equal area or equal distance quantile bins from the range edge). For all categories, edge regions showed reduced relative bird abundance on average compared with non-edge regions. Importantly, ‘non-edge’ does not mean central. Martin *et al*. found that the most central regions do not necessarily have greater abundance than non-central regions, and this depended on whether equal distance or equal area quantiles, or oceanic versus terrestrial edges, were considered. Edge regions had lower abundance on average than non-edge regions in all categories of cases examined, with only a handful of species (mostly coastal species) showing contrasting patterns or no pattern. The magnitude of observations used in this study is hard to match in other types of species (e.g. plants, fungi and rodents), but the findings also only represent birds, which are unique. Birds move perhaps the farthest and easiest of any group of animals. Nonetheless, birds are a diverse group of vertebrates and represent an important case and lineage from which to build an understanding of the biogeography of abundance, and have often served so [1,7].

If edges turn out to be rare in species abundances in many or most cases for other groups of organisms, there are many important implications, including potential causes for what stalls niche evolution and expansion in geographical space, thereby creating species range limits and determining species ranges. Edges are hypothesized to be low in species abundances owing to ecological challenges that limit population size, including increased environmental or biological stress and reduced immigration opportunities [2–4,8]. Pennington *et al*. [9] collectively termed potential effects of strong selection, increased isolation and resulting small population sizes at geographical edges the ‘edge effect’ hypothesis. Abundance may follow few predictable patterns of change from central to edge regions (figure 1*a*) until populations are observed near the edge (figure 1*b*). Step-down and ramp-down (figure 1*c*) patterns of abundance at species range edges have been observed or discussed in studies before (see [10]) and general understanding of such patterns changes over time. For example, Eckert *et al*. [8] found evidence for reduced genetic diversity at species range edges across varying types of organisms, but studies published since have diminished this pattern [5]. In cases where abundance does not ramp down (figure 1*c*), but steps down (figure 1*b*), centres *per se* may have little bearing on the edges; instead, bordering, interior, larger populations may have more important effects on the edge, such as being sources of gene flow, etc. [11]. In cases of abundance declines by ramps or steps, both support the ACH and REH depending on how one defines each (figure 1*c*). Additionally, assuming a strict centre of highest abundance within a species range makes it more likely to reject the ACH, which is one potential reason why the ACH is often found to be untrue even if interior ‘core’ regions of species ranges tend to have higher abundances [7]. Abundant cores and rare edges may be different sides of the same coin [2].

Even if edge populations of species ranges turn out to be mostly very low in occurence or abundance of individuals, it is important to remember that edge populations are often unique and important for their intrinsic, functional and conservation values. As there are so many sizes and shapes of geographical ranges, there are so many types of edges and edge populations. Edges can be regions of genetic novelty, unique adaptations and niche evolution [6], and although edge regions may contain lowest relative abundance on average, not all edge populations are relatively small. Edges can be important strongholds against extinction [12] and harbingers and refugia of geographical range contractions and expansions under changing climates [13]. In many cases, edges are likely to be under-protected (e.g. [14]).

The ACH in the strictest sense has been shown to not be universal, but the related REH has compelling support from Martin *et al*. Refocusing on the REH and turning attention away from birds to other organisms, what will we see? Re-examination of prior data considering the REH is an important next step, and so is more focused sampling of species ranges. Additionally, community science-enabled repositories (e.g. iNaturalist) will allow much greater sampling across more lineages across the tree of life to develop a holistic view of the predictability of species range attributes. Moreover, the results of analyses in Martin *et al*. are specific to the unique biogeographical template of North America. We still know too little about certain regions of Earth, including tropical regions, the Southern Hemisphere and aquatic and marine systems, owing to geographical biases in research. Finally, an important consideration is that many species ranges are currently on the move in response to human-caused climate change. Does this mean we will see more abundantly populated leading edges and less well populated rear or trailing edges through time? If so, understanding geographical abundance patterns also means distinguishing patterns between warm and cold climate edges during times of rapid climate change, like now (figure 1*d*). Martin *et al*. invite future studies to a new chapter of exciting research in ecology, evolution and biogeography.

## Data Availability

This article has no additional data.
